# Age Is a Predictor for the Syncope Recurrence in Elderly Vasovagal Syncope Patients With a Positive Head-Up Tilt Test

**DOI:** 10.3389/fcvm.2021.667171

**Published:** 2021-06-29

**Authors:** Yongjuan Guo, Xiaomin Chen, Tianze Zeng, Lin Wang, Lvwei Cen

**Affiliations:** ^1^Department of Noninvasive Electrocardiology, Ningbo First Hospital, Ningbo, China; ^2^Department of Cardiology, Ningbo First Hospital, Ningbo, China

**Keywords:** age, predictor, syncope recurrence, vasovagal syncope, head up tilt test

## Abstract

**Background:** Valid predictors of the syncope recurrence in vasovagal syncope (VVS) patients with a positive head-up tilt test (HUTT) are currently lacking. The goal of this study was to identify the predictive performance of age for the recurrence of syncope in VVS patients with a positive HUTT.

**Methods:** In total, 175 VVS patients with a positive HUTT were observed for 6–32 months, and the recurrence of ≥1 syncope or typical pre-syncope prodromal episodes during follow-up was considered syncope recurrence. The population was divided into 2 groups, namely, a syncope recurrence group (44 patients) and a no syncope recurrence group (131 patients). The baseline clinical data, haemodynamic parameters, and classification of VVS on the HUTT were analyzed. Logistic regression was used to analyse the effect size and confidence interval for age. A receiver operating characteristic (ROC) curve analysis was used to assess the predictive performance and investigate the predictive value of age by the area under the curve (AUC).

**Results:** The median age of the syncope recurrence group was older than that of the no syncope recurrence group [60.0 (47.8, 66.0) years>53.0 (43.0, 62.0) years], and there was a significant difference between the two groups (*P* < 0.05). The trend for syncope recurrence changed with advancing age, and the logistic regression model adjusted by sex showed that older patients had an increased risk of syncope recurrence in VVS with a positive HUTT [OR value: 1.03, 95% confidence interval (CI): 1.008–1.061, *p* < 0.05]. Age was a valid predictor for the recurrence of syncope in elderly VVS patients with a positive HUTT (AUC: 0.688; 95% CI: 0.598–0.777, *p* < 0.05). The cut-off value was 53.5 years, and the sensitivity and specificity were 72.7 and 52.7%, respectively.

**Conclusions:** Age may be a valid predictor for syncope recurrence in elderly VVS patients with a positive HUTT. The rate of syncope recurrence increased with advancing age, especially in females.

## Background

Syncope is a transient loss of consciousness due to transient global hypoperfusion characterized by rapid onset, short duration, and spontaneous complete recovery. The pre-syncope prodrome includes dizziness, headache, sweating and amaurosis (1). Vasovagal syncope (VVS) is challenging to treat as a heterogeneous disorder. The quality of life and mental health of patients could be seriously affected by recurrent VVS (2, 3). Most clinical studies on the treatment of syncope recurrence in VVS patients were mainly based on head-up tilt test (HUTT) results and the VVS classification. However, results of these studies have been inconsistent (4–8). Severe and refractory syncope is usually treated with medical or device therapy. Thus, valid prediction of recurrent syncope is urgently needed to guide the choice of treatment options. Factors that can best predict syncope recurrence after a positive HUTT are few despite numerous randomized trials. Sheldon et al. found that the most powerful predictor of syncope recurrence was the logarithm of the number of preceding syncopal spells (9). We attempted to identify whether age is an important factor affecting syncope recurrence in VVS patients.

## Methods

### Subjects

**Figure 1:**
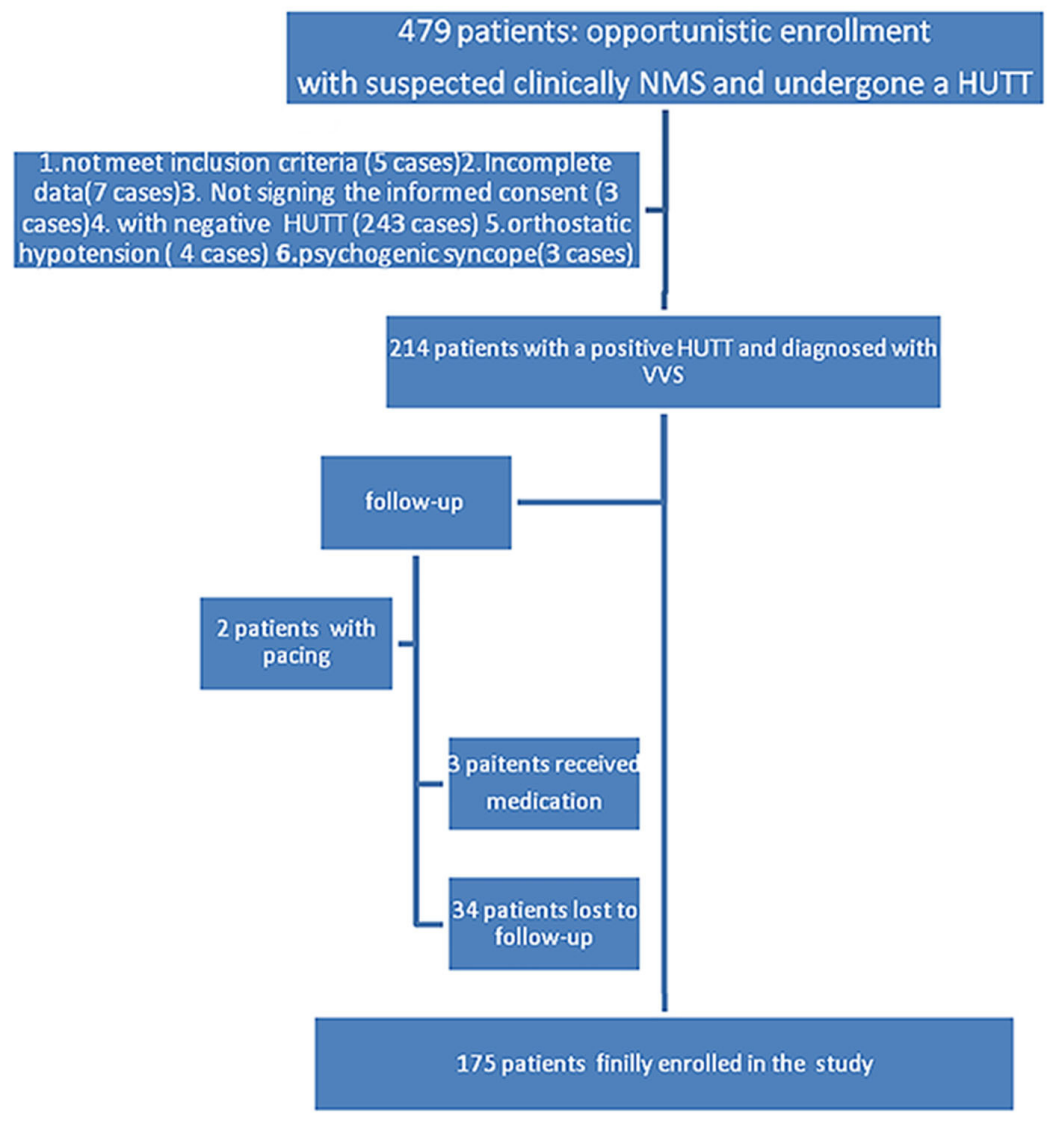
A study flowchart with included and excluded patients.

In total, 479 inpatient and outpatients who were clinically suspected neuro-mediated syncope (NMS) underwent a HUTT in Ningbo Hospital, Zhejiang University from April 2016 to June 2018, which was performed in accordance with the 2018 ESC Guidelines for the diagnosis and management of syncope (1); All patients underwent a complete physical examination, chest X-ray, biochemical serum tests, echocardiography, electrocardiogram (ECG) or Holter (if necessary), etc. to exclude severe diseases related to the central nervous, cardiovascular, and metabolic systems, such as sinus node or conduction system disease etc. Two hundred sixty-five patients were excluded due to not meet inclusion criteria (5 cases), incomplete data (7 cases), not signing the informed consent (3 cases), with a negative HUTT (243 cases), orthostatic hypotension (4 cases), or psychogenic syncope (3 cases) who were not tilted for the full 45 min period (Figure 1). A total of 214 inpatients and outpatients with a positive HUTT were diagnosed with VVS. Then, they were followed up for between 6 and 32 months. Three patients were treated pharmacologically (small doses of metoprolol or theophylline drugs, per os), 2 patients received pacemaker treatment (just DDD pacemaker), and 34 patients were lost to follow-up (Figure 1). The data for 175 patients with a median age of 55.0 years [interquartile range: 44.0, 63.0 years, range from 15 to 82 years] were finally enrolled in the analysis; data from patients who were lost to follow-up or received medication or pacing were excluded from the analysis. The study was approved by the ethics committee of Ningbo First Hospital and carried out in accordance with the Declaration of Helsinki.

### HUTT (1)

#### Preparation

The HUTT was performed strictly in accordance with the protocol of the European Society of Cardiology. Cardiovascular active drug treatments were stopped for at least a 5 half-life period. Drugs and foods that could have affected normal autonomic nervous system function were avoided before the test. The HUTT was performed in a quiet, softly lit, temperature-controlled (20~25°C) room equipped with medical resuscitation facilities, such as a defibrillation apparatus and atropine and other resuscitative drugs. The patients were fasted for at least 4 h before the HUTT. Basic clinical data related to cerebrovascular diseases (including cerebral ischemia, cerebral infarction, epilepsy, etc.), hypertension, cardiovascular disease, diabetes (including type 1 or type 2), natural course of disease and number of spells in lifetime was needed to be available for inclusion in the study.

#### Baseline HUTT

The patients were secured to the electric head-up tilt test table with a manual control board (ST-721, Beijing Juchi Medical Technology Co. Ltd), and a 12-lead synchronous ECG monitor (CASE, General Electric Company) and an automated sphygmomanometer (Tango M2, SunTech Medical Inc) was used for continuous monitoring of heart rate, rhythm, and blood pressure. Barring a positive response or loss of consciousness, the patients were asked to lie supine for at least 10 min with supine systolic blood pressure, supine diastolic blood pressure, and heart rate recorded, and then the table was tilted to 70°. The HUTT was terminated at any time when a positive reaction or orthostatic hypotension or psychogenic syncope was occurrence, or the patient requested to terminate because of intolernce; Otherwise, the tilted position would be maintained for full 45 min. Blood pressure, heart rate, and rhythm were recorded every 5 min and every 1 min in the initial 3 min to rule out orthostatic hypotension during the test. Symptoms were recorded in real-time. The patients were returned to a supine position as soon as a positive response occurred. The test was terminated if the HUTT was positivein the basic stage; otherwise, the test was continued with the sublingual nitroglycerin HUTT.

#### Sublingual Nitroglycerin HUTT

The process of sublingual nitroglycerin HUTT (SNHUTT) was the same as that in the basic stage assessment but followed the administration of sublingual nitroglycerin (300–400 μg, 0.5 mg/tablet of Xinyi Pharmaceutical Co., Ltd.) in the baseline HUTT-negative patients.

### Diagnostic Criteria

Syncope or pre-syncope prodrome accompanied by any decreases in blood pressure or changes in heart rate with an electrocardiogram showing sinus arrest, nodal or ventricular escape, atrioventricular block or cardiac arrest ≥3 s was a positive HUTT response characteristic.

### Positive VVS Response Types

#### Type 1 (Mixed Type)

Type 1 is characterized as a heart rate fall but not to <40 beats per minute (bpm) for <10 s at the time of syncope with or without asystole <3 s. The blood pressure decrease occurs prior to the heart rate fall.

#### Type 2 (Cardioinhibitory Type) Is Classified Into 2 Subtypes

##### Type 2A (Without Asystole)

Type 2A is characterized as a ventricular rate below 40 bpm for longer than 10 s and asystole <3 s. The blood pressure decrease occurs later than the heart rate fall.

##### Type 2B (With Asystole)

Type 2B is characterized as asystole >3 s. The heart rate fall coincides with or precedes the blood pressure decrease.

#### Type 3 (Vasoinhibitory Type)

Type 3 is characterized as systolic blood pressure (SBP) or mean pressure decrease ≥20 ~ 30 mmHg or SBP ≤ 60 ~ 80 mmHg (1 mmHg = 0.133 kPa). The heart rate does not fall more than 10% from its peak value at the time of syncope.

### Counseling and Advice (Conservative Treatment)

All patients with a positive HUTT were provided with an overview of the VVS cause and its overall benign outcome and coached on how to avoid some situations that might provoke syncope, such as fatigue, late nights, particular emotional states, etc. They were also asked to sit down or lie in a supine position if syncope was unavoidable. They also received advice about increasing dietary salt and fluid intake unless contraindicated.

### Follow-Up Protocols

The duration of follow-up ranged from 6 to 32 months after the HUTT by individual telephone calls to all patients. The patients or their parents were asked about syncope recurrence, which was defined as the recurrence of ≥1 syncope or typical pre-syncope prodrome that occurred during the follow-up. Patients who underwent pacing or related drug therapy, such as metoprolol treatment, were excluded during the follow-up study.

### Statistical Analysis

All reported levels of significance are 2 sided. A *P* ≤ 0.05 was considered statistically significant. Statistical analysis was carried out with SPSS 23.0 software. Continuous variables with normal distributions are expressed as the mean ± standard deviation (SD), and comparisons of normally distributed parameters between the two groups were performed with a *t*-test for independent samples. The non-normally distributed parameters are reported as the median and interquartile range (25–75%) and compared by the Mann-Whitney *U*-test. Categorical variables are reported as frequencies and percentages. The data were compared by Pearson's chi-square test, and the exact probability method was used when the theoretical frequency was <20%. Logistic regression was used to analyse the effect size and confidence interval of the individual or multiple factors with statistical significance for syncope recurrence based on the independent sample *t*-test or Pearson's chi-square test. The predictive performance of age was evaluated by prediction probability. The receiver operating characteristic (ROC) curve was utilized to evaluate the predictive value of the predictors, and the area under the curve (AUC) represented the predictive value. A 95% CI of the AUC that did not contain 0.5 or a *P* < 0.05 confirmed that the factor was a reliable predictor of recurrent syncope in VVS patients with a positive HUTT. The optimal cut-off value was determined as the maximum Youden index, which was defined as the sensitivity plus specificity minus 1, where sensitivity and specificity were calculated as proportions.

## Results

### Patient Population

The valid data of 175 VVS patients with a positive HUTT were included in the analysis. Forty-four (25.1%) VVS patients had ≥1 recurrence of syncope or typical pre-syncope prodrome during follow-up.

### Baseline Characteristics

The baseline characteristics of the two groups are shown in Table 1. The syncope recurrence group was older than the no syncope recurrence group (*P* < 0.05). Other characteristics were not significantly different between the groups (*P* > 0.05).

**Table 1:** Baseline characteristics of the study population and follow-up duration.

**Cases (*n* = 175)**	**Syncope recurrence group**	**No syncope recurrence group**	**t/*X*^**2**^/*Z*-value**	***P*-value**
	**(*n =* 44)**	**(*n =* 131)**		
Males/Females (*n*)	13/31	58/73	2.964	0.085
Age (yrs)	60.0 (47.8, 66.0)	53.0 (43.0, 62.0)	−2.346	0.019
BMI (kg/m2)	23.3 ± 3.1	22.4 ± 2.9	1.622	0.107
**LVEF (%)**	**60.4** **±** **4.7**	**61.4** **±** **5.4**	**1.100**	**0.273**
**LVDd (mm)**	**44.9** **±** **2.9**	**44.1** **±** **2.8**	–**1.618**	**0.107**
Patients with comorbidities (*n*)	19 (43.2%)	49 (37.4%)	0.463	0.496
Hypertension (*n*)	11 (25.0%)	36 (27.5%)	0.103	0.748
Cardiovascular disease (*n*)	3 (6.8%)	7 (5.3%)	0.000	1.000
Diabetes (*n*)	3 (6.8%)	4 (3.1%)	0.433	0.511
Cerebrovascular disease (*n*)	1 (2.3%)	3 (1.5%)	0.000	1.000
**Calcium channel blockers**	**6 (13.6%)**	**13 (9.9%)**	**0.164**	**0.686**
**Diuretics**	**3 (6.8%)**	**10 (7.6%)**	**0.000**	**1.000**
**ACE-I/ARB**	**5 (11.4%)**	**11 (8.4%)**	**0.083**	**0.773**
**β-blockers**	**3 (6.8%)**	**7 (5.3%)**	**0.000**	**1.000**
**Nitrates**	**2 (4.5%)**	**4 (3.1%)**	**0.000**	**1.000**
Duration of symptoms (<2 years)	25 (56.8%)	84 (64.1%)	0.748	0.387
Number of spells in lifetime (more than 3 times)	22 (50.0%)	52 (39.7%)	1.433	0.231
Follow-up duration (months)	18.0 (14.0, 25.0)	16.0 (11.0, 23.0)	−1.482	0.138

### HUTT Characteristics

The HUTT characteristics of the two groups are shown in Table 2. None of the HUTT characteristics were significantly different between the groups (*P* > 0.05).

**Table 2:** Baseline positive HUTT characteristics of the study population.

**Cases (*n =* 175)**	**Syncope recurrence group**	**No syncope recurrence group**	***t*/*X*^**2**^-value**	***P*-value**
	**(*n =* 44)**	**(*n =* 131)**		
Positive BHUTT/Positive SNHUTT	5/39	6/125	1.550	0.213
HR (bpm)	71.1 ± 15.2	70.47 ± 12.1	0.263	0.793
Supine systolic BP (mmHg)	126.3 ± 19.1	124.7 ± 18.8	0.482	0.630
Supine diastolic BP (mmHg)	78.3 ± 9.9	75.5 ± 11.2	1.469	0.144
Supine heart rate (bpm)	71.1 ± 15.2	70.5 ± 12.1	0.263	0.793
Induced Syncope (*n*)	19	58	0.016	0.899
Arrhythmic events (*n*)	4	18	0.648	0.421
Type 1 (mixed type) (*n*)	19	69	1.187	0.276
Type 2 (cardioinhibitory type) (*n*)	3	10	0.000	1.000
Type 2A (*n*)	1	5	0.000	0.993
Type 2B (*n*)	2	5	0.000	1.000
Type 3 (vasodepressor type) (*n*)	22	52	1.433	0.231

### Distribution of Patients by Months of Follow-Up

**Figure 2:**
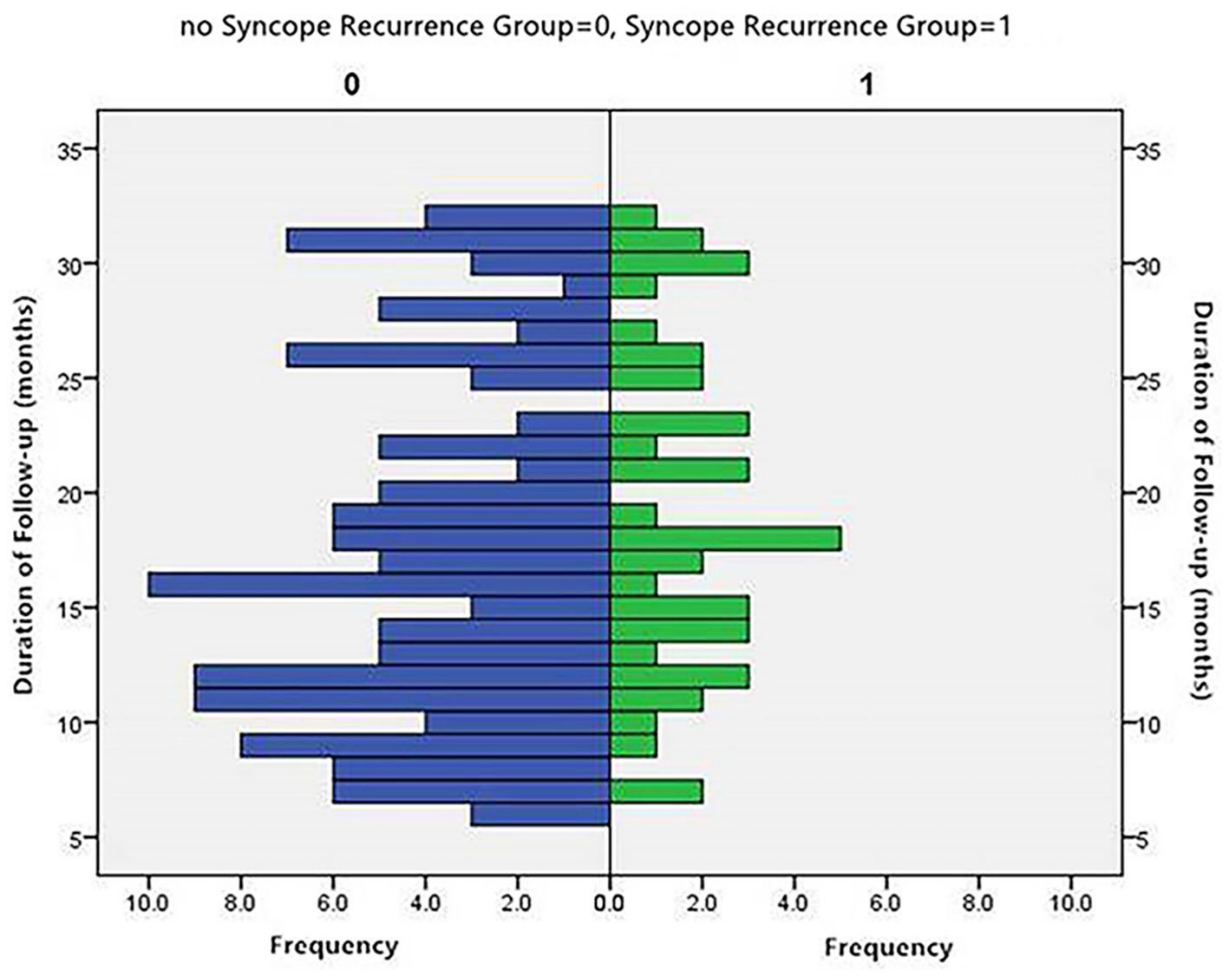
Distribution of patients by months of follow-up.

The distribution of patients by month of follow-up is shown in Figure 2. The distribution was inconsistent between the two groups. The average rank of the data shown in Table 2 was not significantly different between the groups (*P* > 0.05).

### Trend in Syncope Recurrence Changed With Advancing Age

**Figure 3:**
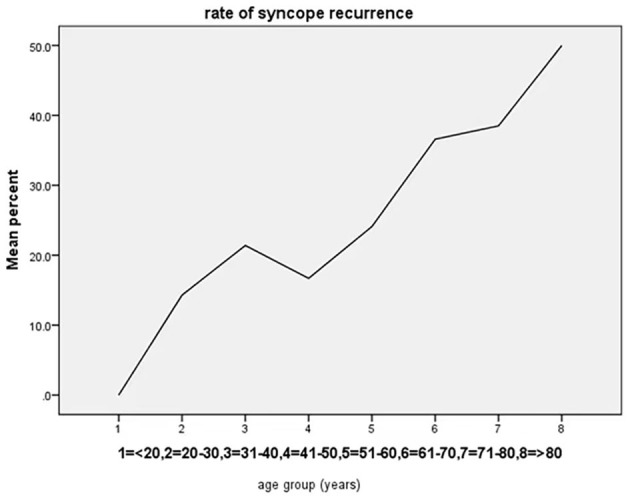
Age-trend for syncope recurrence based on the percentage within each age group. The rate of syncope recurrence increased with advancing age.

The study population was classified based on age into 8 groups (<20, 20–30, 31–40, 41–50, 51–60, 61–70, 71–80, and >80 years old), and the graph shows that the rate of syncope recurrence increased with advancing age (Table 3, Figure 3).

**Table 3:** Sample sizes for all ages in the study population.

**Age (years)**	** <20**	**20–30**	**31–40**	**41–50**	**51–60**	**61–70**	**71–80**	**>80**
Recurrence (*n*)	0	2	3	5	13	15	5	1
No recurrence (*n*)	7	12	11	25	41	26	8	1
Percent (%)	0	14.3	21.4	16.7	24.1	36.6	38.5	50

### Predictive Performance of Age

Age, sex, BMI, and supine diastolic BP (all *p* < 0.15 in the univariate analysis) were included in the logistic regression analysis. Only age, which was statistically significant in the univariate analysis, (OR value: 1.034, *p* = 0.011) and sex (OR value: 2.302, *p* = 0.032) were independently associated with syncope recurrence in VVS with a positive HUTT in the multivariable model (Table 4).

**Table 4:** Determinants of syncope recurrence in the study population.

**Factor**	**B value**	**S.E value**	**Wald value**	***P*-value**	**Exp(B)**	**95%CI of Exp(B)**
Sex	0.834	0.389	4.598	0.032	2.30	1.074–4.932
Age	0.034	0.013	6.427	0.011	1.03	1.008–1.061

*CI, confidence interval*.

### Predictive Ability of Age According to the ROC Curve Analysis

**Figure 4:**
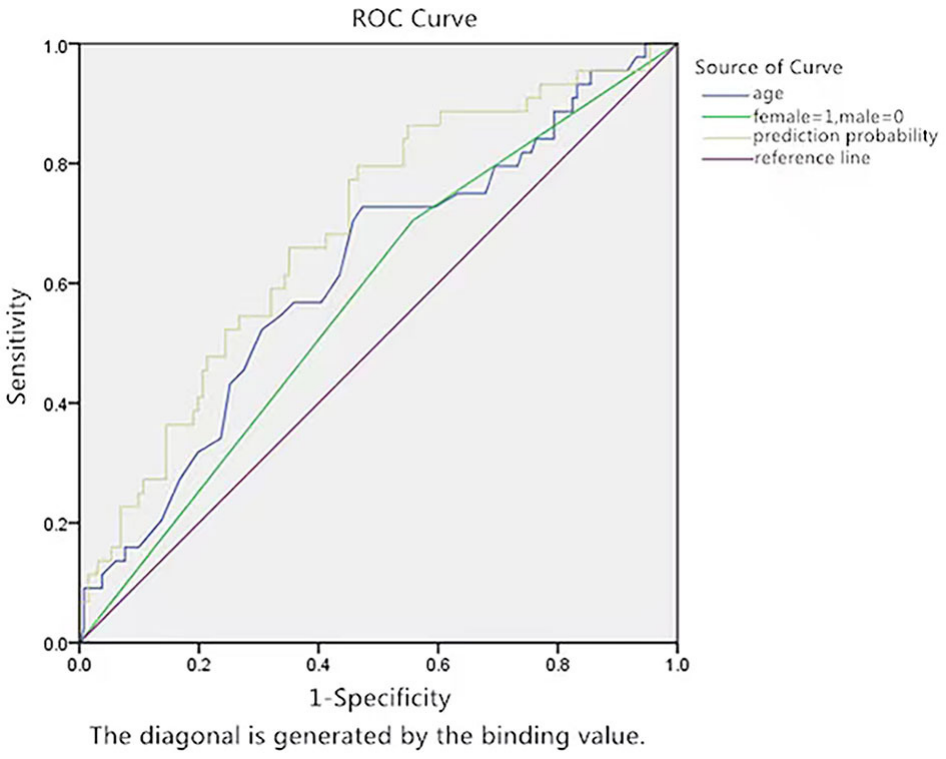
ROC curve of the predictive value of age for syncope recurrence by prediction probability. The y-axis represents the sensitivity to predict syncope recurrence. The x-axis represents the false positive rate (1-specificity) of prediction. The green line in the graph is the reference line, which indicates that the sensitivity is equal to the false positive rate. The blue curve is farther from the green line and nearer to the upper left corner of the graph. The area under the curve was 0.688 (95% CI: 0.598–0.777, *P* < 0.05).

The ROC curve of age for the prediction of syncope recurrence in VVS patients with a positive HUTT had an AUC of 0.688 (95% CI: 0.598–0.777, *p* < 0.05; vs. the null hypothesis AUC of 0.5). A cut-off value of 53.5 years of age yielded high sensitivity (72.7%) and specificity (52.7%) (Figure 4).

## Discussion

The main findings of this study are as follows: (1) the total rate of syncope recurrence was 25.1% (44/175 cases) in VVS patients (median age: 55.0 years) with a positive HUTT, and it increased with advancing age during follow-up in our study. (2) We found a significant relationship between age and syncope recurrence. Elderly patients had an increased risk of syncope recurrence in VVS with a positive HUTT, and the cut-off value was 53.5 years, especially in females. Age as a predictor of syncope recurrence fulfilled all 3 criteria for the highest level of statistical significance according to single factor analysis, binary logistic regression, and receiver operating characteristic (ROC) curve analysis. In contrast, other factors, such as Vasovagal Syncope International Study (VASIS) classification and the number of syncope episodes, were not statistically significant.

VVS with atypical features is often diagnosed by a HUTT and is known to have a benign prognosis; however, recurrent VVS can seriously affect the quality of life and mental health of patients, particularly older adults who usually experience atypical VVS, which may have little or no prodrome (10). Therefore, valid predictors for the recurrence of syncope in VVS, especially in VVS patients with a positive HUTT, are very important for the design of efficient, economical, individualized treatment approaches. Sumner et al. proposed that the number of syncope episodes in the year preceding presentation was the most powerful predictor of the time to the recurrence of syncope in a referral-based VVS population (11). There was a similar phenomenon in which the rate of ≥3 lifetime syncopal spells in the syncope recurrence group tended to be higher than that in the no syncope recurrence group (50.0 vs. 39.7%, respectively) in our study, although this difference was not significant. Another important finding was that age adjusted by sex was significantly associated with syncope recurrence. The results indicated that agedness was a valid predictor of syncope recurrence in VVS patients with a positive HUTT.

Previous reports have focused their attention on the research of patient history, the number of preceding syncopal spells, arterial baroreflex sensitivity, serum biochemical parameters, etc. (9, 12, 13). HUTT is usually used to define the syncope recurrence in those reports. In this study, we enrolled older patients and defined recurrence of syncope in nature state rather than HUTT. In addition, the patients enrolled in this study received professional guidance from the clinician including ensuring sufficient sleep, the relief from anxiety and fatigue, avoiding exciting habits, and activities and proper response measures in the event of pre-syncope. All of those could help to reduce the syncope recurrence in patients with VVS.

The results may be attributed to the following, although the reason for this is not fully clear:

First, autonomic control of the cardiovascular system could be influenced by both age and sex (14, 15). Maria et al. found attenuation of the autonomic nervous system (ANS) in the regulation of cardiac function in older essential hypertensive patients (16), and Benditt et al. discovered a lower epinephrine (Epi)/norepinephrine (NE) ratio in older patients than in younger patients with syncope during tilt-induced VVS (17). NE is the junctional transmitter between sympathetic ganglion cells and effectors. Indeed, a decrease in cardiac sympathetic and parasympathetic components was observed in the older population. The autonomic control of blood pressure appears to decline with aging (18). These observations could reflect a decreased sensitivity of cardiac beta-adrenoceptors with aging. The effect of aging might be related to the parallel age-related reduction in sympathetic vasomotor responsiveness. Attenuation of this process results in autonomic nerve function damage, and ANS imbalance may be one of the underlying mechanisms (19).

Second, in adults, a decrease in cardiac output (CO) is the dominant hypotensive mechanism, and it is not vasodilatation because systemic vascular resistance (SVR) always remains above baseline levels during VVS (20). Yamaguchi et al. proposed that among patients with a positive HUTT, the syncope recurrence rate after the HUTT in those with LV dysfunction was higher than that in those with normal LV function (21), and relatively older patients often have LV dysfunction because of cardiovascular comorbidities. Interestingly, our study also showed that the rate of comorbidities in the syncope recurrence group tended to be higher than that in the no syncope recurrence group (43.2 vs. 37.4%, respectively), although the difference was not significant.

In addition, sustained orthostatic hypotension and delayed BP recovery are more common and are risk factors for falls, injuries, and cognitive decline in the older population. Hence, a mild orthostatic hypotension response during orthostatic stimulation may take part in syncope recurrence despite being diagnosed with VVS in the HUTT.

Based on our findings, recurrent syncope is more common in older patients with a positive HUTT, but that the likely results from multiple mechanisms, not just VVS. Greater clinical vigilance and other considerations may effectively decrease the frequency of syncope recurrence in old VVS patients (older than 53.5 years) with a positive HUTT, such as continued antihypertensive therapy and active treatment of ventricular dysfunction.

### Limitations

The present study also has limitations. First, our study was an open-label, observational, and single-center retrospective study, and selection bias is inevitable. Second, the inability to know the cause of recurrent syncope, detailed confounding comorbidities in the older patients and the lack of data on LV function at the time of recurrent syncope resulted in the inability to stratify the population. Third, the interval from the HUTT to the first recurrence of syncope was observed during follow-up, and the duration of follow-up varied. In addition, there are known limitations to the AUC statistical method. Randomized controlled studies are essential to assess the predictive value of age.

## Conclusions

Age may be a valid predictor for the recurrence of syncope in elderly VVS patients with a positive HUTT. The rate of syncope recurrence increased with advancing age, especially in females. VVS patients who were older than 53.5 years with a positive HUTT had a greater recurrence possibility despite receiving non-pharmacological measures other than pacing. This intriguing novel finding deserves further study.

## Data Availability Statement

The raw data supporting the conclusions of this article will be made available by the authors, without undue reservation.

## Ethics Statement

The studies involving human participants were reviewed and approved by the ethics committee of Ningbo First Hospital. Written informed consent to participate in this study was provided by the participants' legal guardian/next of kin.

## Author Contributions

YG participated in the design of the study, data collection, and drafting of the manuscript. XC conceived the study and participated in its design. TZ, LW, and LC participated in the design of the study and data collection. All authors read and approved the final manuscript.

## Conflict of Interest

The authors declare that the research was conducted in the absence of any commercial or financial relationships that could be construed as a potential conflict of interest.

## Abbreviations

VVS: vasovagal syncope
HUTT: head-up tilt test
BHUTT: baseline HUTT
SNHUTT: sublingual nitroglycerin HUTT
ROC: receiver operating characteristic
AUC: area under the curve
ANS: autonomic nervous system
Epi: epinephrine
NE: norepinephrine
CO: cardiac output
SVR: systemic vascular resistance.
